# Maternal HIV infection and the milk microbiome

**DOI:** 10.1186/s40168-024-01843-8

**Published:** 2024-09-28

**Authors:** Nicole H. Tobin, Fan Li, Sean Brummel, Patricia M. Flynn, Sufia Dababhai, Dhayendre Moodley, Lameck Chinula, Avy Violari, Mary Glenn Fowler, Vanessa Rouzier, Louise Kuhn, Grace M. Aldrovandi, for the GUMBO, ZEBS, 1077BF/1077FF PROMISE Teams

**Affiliations:** 1 Division of Infectious Diseases, Department of Pediatrics, David Geffen School of Medicine at the University of California, 10833 Le Conte Ave., 22-340 MDCC, Los Angeles, CA 90095 USA; 2 The Center for Biostatistics in AIDS Research, Harvard T.H. Chan School of Public Health, Boston, MA USA; 3 Department of Infectious Diseases, St. Jude Children’s Research Hospital, Memphis, TN USA; 4 Johns Hopkins Bloomberg School of Public Health, Blantyre, Malawi; 5 Centre for the AIDS Programme of Research, School of Clinical Medicine, University of KwaZulu Natal, Durban, South Africa; 6 University of North Carolina (UNC) Project Malawi, Lilongwe, Malawi; 7 Perinatal HIV Research Unit, University of the Witwatersrand, Johannesburg, South Africa; 8 Department of Pathology, Johns Hopkins University School of Medicine, Baltimore, MD USA; 9 The Haitian Group for the Study of Kaposi’s Sarcoma and Opportunistic Infections, GHESKIO Centers, Port-Au-Price, Ouest 15727 Haiti; 10 Gertrude H. Sergievsky Center, Vagelos College of Physicians and Surgeons, New York, NY USA; 11 Department of Epidemiology, Mailman School of Public Health, Columbia University Irving Medical Center, New York, NY USA

**Keywords:** Human breast milk microbiome, HIV-1, HIV, HEU, CHEU, HIV-exposed uninfected, Breast milk transmission, Infants, Children, Lactation

## Abstract

**Background:**

Children born to women with HIV but who do not become HIV infected experience increased morbidity and mortality compared with children born to women without HIV. The basis of this increased vulnerability is unknown. The microbiome, specifically the infant gut microbiome, likely plays an important role in infant immune development. The human milk microbiome is thought to have an important role in the development of the infant gut and therefore, if perturbed, may contribute to this increased vulnerability. We investigated the effects of HIV and its therapies on the milk microbiome and possible changes in the milk microbiome before or after infant HIV infection.

**Results:**

Seven-hundred fifty-six human milk samples were selected from three separate studies conducted over a 15-year period to investigate the role of HIV and its therapies on the human milk microbiome. Our data reveal that the milk microbiome is modulated by parity (*R*^2^ = 0.006, *p* = 0.041), region/country (*R*^2^ = 0.014, *p* = 0.007), and duration of lactation (*R*^2^ = 0.027–0.038, all *p* < 0.001). There is no evidence, however, using 16S rRNA V4 amplicon sequencing, that the human milk microbiome is altered by HIV infection (*R*^2^ = 0.003, *p* = 0.896), by combination antiretroviral therapy (*R*^2^ = 0.0009, *p* = 0.909), by advanced maternal disease (*R*^2^ = 0.003, *p* = 0.263), or in cases of infant infection either through isolated early mucosal (*R*^2^ = 0.003, *p* = 0.197) or early mucosal and breast milk transmission (*R*^2^ = 0.002, *p* = 0.587).

**Conclusions:**

The milk microbiome varies by stage of lactation, by parity, and by region; however, we found no evidence that the human milk microbiome is altered by maternal HIV infection, disease severity, or antiretroviral therapy. Additionally, we found no association between the milk microbiome and transmission of HIV to the infant. Investigations including higher resolution microbiome approaches or into other potential mechanisms to understand why the approximately one million children born annually to women with HIV escape infection, but do not escape harm, are urgently needed.

Video Abstract

**Supplementary Information:**

The online version contains supplementary material available at 10.1186/s40168-024-01843-8.

## Background

The role of the microbiome in HIV is controversial. Some studies have reported differences in the microbiome associated with HIV infection, while others have not [1, 2]. Behavior and other factors have emerged as significant variables contributing to these discrepancies [2, 3]. The human milk microbiome is believed to play an important role in the development of the infant gut microbiome although the relationships are yet to be clearly defined [4]. However, there are data that the infant gut microbiome is critical for infant immune development and health [4, 5].

Children born to women with HIV (WWH) who do not acquire HIV infection themselves, often referred to as children who are HIV-exposed, uninfected (CHEU), experience increased morbidity and mortality compared with children born to women without HIV (WWoH); children whose mothers do not have HIV infection are called HIV-unexposed, uninfected (CHUU) [6, 7]. The increased morbidity in CHEU is related to both viral and bacterial infections with a 12-fold increase in mortality from respiratory syncytial virus pneumonia in the first 6 months of life [8] as well as an increased risk of serious bacterial infections [9]. In the United States, CHEU have twice the hospitalization rate of CHUU [10]. Additionally, a large meta-analysis demonstrated that CHEU have a 50% increase in mortality [11]. Initiation of antiretroviral therapy (ART) before pregnancy reduces infection-related hospitalizations of CHEU [12]. One study suggests a relationship between innate immune responses and the gut microbiome in CHEU, with the caveat that the relationships are specific to different populations [13]. CHEU also suffer from stunting of growth and appear to be at increased risk of neurodevelopmental delays [6].

There is also emerging evidence that ART may induce changes in the microbiome independent of the effects of HIV-infection [14]. The potential mechanisms by which ART may alter the human milk microbiome include direct antibacterial effects of ART on milk microbes and on the maternal gut microbes. Alterations in maternal gut microbes may shape the microbes transferred to the milk through an enteromammary pathway [15]. A few studies demonstrate that specific antiretrovirals may have direct antimicrobial effects [14, 16–18]. Zidovudine and efavirenz have in vitro antibacterial activity against *Bacteroides fragilis* and *Prevotella*, zidovudine and 2’,2’-dideoxyinosine inhibit the growth of *Escherichia coli*, and efavirenz also inhibits the growth of *Enterococcus faecalis* and *Bacillus subtilis* [14, 16]. Protease inhibitors may decrease the ability of *Candida albicans* to adhere to endothelial cell layers, [17] and maraviroc is associated with decreased Enterobacteriales in mice on a high fat diet [18]. Given the possible effects of (1) differing ART regimens on the milk microbiome, (2) the potential importance of the milk microbiome on HIV transmission, and (3) the potential importance of the milk microbiome on normal gut-associated immune development of the infant, it is important to determine the effects of differing ART regimens on the human milk microbiome that may subsequently affect the infant gut.

Since CHEU experience high rates of morbidity and mortality and human milk plays a crucial role in infant health, we sought to investigate effects of HIV and its therapies on the human milk microbiome. Additionally, we sought to explore whether there were changes in the maternal milk microbiome prior to or following infant HIV infection.

## Methods

### Clinical trial groups, inclusion and exclusion criteria, and parent trial study intervention

**Fig. 1:**
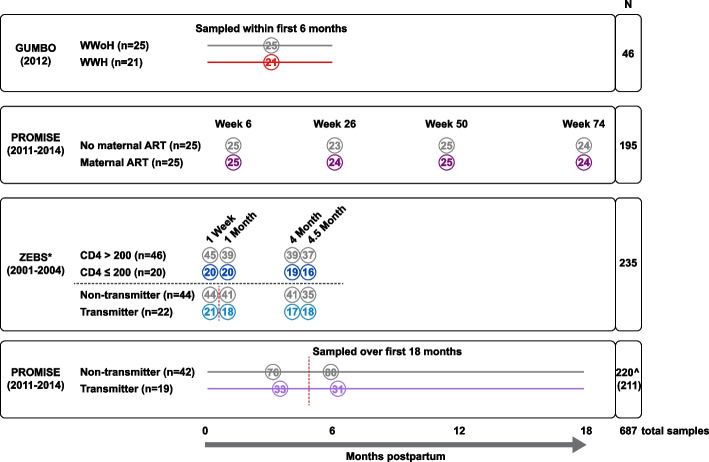
Study schematic of participants and samples included in the final analyses. Overall study design and distribution of samples from each cohort. Each rectangular box indicates a sub-study, with the number of participants included in the final analyses indicated in parentheses next to each grouping variable. The open circles denote the timing and number of samples included in the final analysis. The dotted vertical lines show the best estimate for timing of mother-to-child transmission of HIV in the transmitters and timing of pre- and post-samples for comparison in the non-transmitters. The total number of samples is the number of samples included in the final analyses. *ZEBS samples were analyzed to assess advanced maternal disease as well as cases of transmission. ^Parentheses in total sample number (*N*) column for PROMISE transmission cohort is the number of unique samples, 9 samples from 4 participants were also part of the ART analysis. *WWoH* women without HIV, *WWH* women with HIV, *ART* antiretroviral therapy

Seven-hundred fifty-six human milk samples were selected from three separate studies to investigate the role of HIV-1 and its therapies on the human milk microbiome: a study in Haiti called GUMBO (GUt Microbiome, Breastmilk and Oligosaccharides) [19], the International Maternal Pediatric Adolescent AIDS Clinical Trials Network PROMISE (Promoting Maternal Infant Survival Everywhere) Trial [20, 21], and the Zambia Exclusive Breastfeeding Study (ZEBS) [22] (see Fig. 1).

### Sample selection and comparison groups

In GUMBO (2012) [19], women in Haiti on maternal ART who had delivered in the previous 6 months and were primarily breastfeeding were eligible to enroll in this cross-sectional study of 25 WWH and 25 WWoH. Milk samples from 24 WWH whose infants were HIV negative at the time of sample collection and 25 WWoH were selected (49 samples total).

In PROMISE [20, 21], postpartum mother-infant pairs, where the mother had a CD4 count = or > 350 and did not meet country specific treatment guidelines at the time of the study (2011–2014), were randomized to receive either maternal ART with tenofovir, emtricitabine, and ritonavir-boosted lopinavir or infant nevirapine (no maternal ART) for prophylaxis of breast milk transmission for the duration of breastfeeding. All infants received nevirapine for the first 6 weeks of life to prevent intrapartum transmission. To determine if maternal ART altered the milk microbiome, longitudinal maternal milk samples at 6, 26, 50, and 74 weeks postpartum were selected from 25 women randomized to maternal ART and 25 women whose infants received nevirapine for prophylaxis where transmission did not occur. These participants were matched on baseline maternal CD4 count, viral load, country, and date of study randomization.

To determine if the human milk microbiome was altered prior to or following transmission of HIV in the setting of antiretroviral prophylaxis, 4 milk samples per participant in the PROMISE cohort were selected prior to and following the transmission event. Nine samples from 4 participants in the PROMISE maternal ART cohort were included as controls in this analysis. As transmission of HIV can occur *in utero,* intrapartum or via human milk, the transmission events were defined based on the time that the infant first tested positive for HIV infection. In PROMISE, early mucosal transmission, where intrapartum and breast milk transmission cannot be distinguished, was defined as a negative infant HIV PCR in the first 14 days of life followed by the next sample available testing positive. Breast milk transmission was defined as a negative infant HIV PCR at 6 weeks followed by a positive sample later during the course of the study. Twenty-one women with early mucosal or breast milk transmission cases were matched on maternal baseline CD4 count, HIV plasma RNA levels (viral load), date of study randomization, and days of lactation at a 1:2 ratio with 42 WWH whose infants were not infected with HIV (252 samples total).

ZEBS [22] was conducted in Lusaka, Zambia, between 2001 and 2004, prior to the standard use of maternal antiretroviral therapy. The women in the study received single-dose nevirapine at the initiation of labor for the prevention of mother-to-child transmission of HIV as was the standard of care at the time. To investigate the effect of advanced maternal disease on the milk microbiome, we evaluated the milk of 20 women with a baseline CD4 T-cell count of < or = 200 cells/mm^3^ versus 46 women with > 200 cells/mm^3^ (264 samples total).

To determine the role of the human milk microbiome in early mucosal transmission in ZEBS, infants who tested DNA PCR negative for HIV at 1 week of life, but positive at 1 month of life, were selected. Four longitudinal milk samples per participant were selected prior to and following detectable infant infection. Longitudinal human milk samples from 22 early mucosal transmission cases and 44 control WWH matched 1:2 on maternal viral load and CD4 count were selected from 1 week, 1 month, 4 months, and 4.5 months of lactation (same 264 samples as analyzed by CD4 count).

### Sample collection

In all 3 studies, participants and staff washed their hands and in PROMISE washed their breast(s) with water or soap and water before milk samples were collected at study visits. In GUMBO, PROMISE, and for the majority of the ZEBS samples, the milk was manually expressed into sterile containers. The first few drops were discarded in GUMBO and ZEBS before collection. In ZEBS, a milk pump was offered, but infrequently used. In GUMBO and ZEBS, aliquots of whole milk were transferred on “wet” ice and stored within 2 h at − 20 to − 70 °C initially and then transferred to permanent storage at − 70 °C. In PROMISE, milk was placed on “wet” ice within 10 min of collection. Milk was kept cold and aliquoted within 4–6 h of collection and stored at − 70 °C.

### 16S rRNA Sequencing

The PROMISE and ZEBS samples were processed at the same time in the CHOP Microbiome Center at the Children’s Hospital of Philadelphia. DNA was extracted from approximately 200 uL of human milk using the Qiagen DNeasy PowerSoil Pro kit (Germantown, Maryland, USA). Extracted DNA was quantified with the Quant-iT PicoGreen Assay Kit (Waltham, MA, USA). PCR amplification of the V4 region of 16S rRNA gene was performed with 515F/806R primers in duplicate using Q5 High-Fidelity DNA Polymerase (NEB, Ipswich, MA). Each PCR reaction contained 0.5 uM of each primer, 0.34 U Q5 Pol, 1X Buffer, 0.2 mM dNTPs, and 10.0 ul DNA in a total volume of 50 ul. Cycling conditions were as follows: 1 cycle of 98 °C for 1 m; 20 or 25 cycles of 98 °C for 10 s, 56 °C for 20 s, and 72 °C for 20 s; and 1 cycle of 72 °C for 8 m. After amplification, duplicate PCR reactions were pooled and then purified using a 1:1 volume of SPRI beads. DNA in each sample was then quantified using PicoGreen and pooled in equal molar amounts. The resulting library was sequenced on the Illumina MiSeq using 2 × 250 bp chemistry. Extraction blanks and DNA free water were subjected to the same amplification and purification procedure to allow for empirical assessment of environmental and reagent contamination. Positive controls, consisting of five artificial 16S gene fragments synthesized in gene blocks and combined in known abundances, were also included. The GUMBO samples were processed in the Aldrovandi Laboratory in 2014–2015 by centrifugation of 1.5 mL of whole milk, resuspending the pellet, and extracting alongside PCR water and extraction buffer negative and a bacterial mock community positive controls as previously described [19, 23].

### Data processing and statistical analysis

Dada2 (v1.22.0) [24], decontam (v.1.14.0) [25], and phyloseq (v1.38.0) [26] were used for sequence inference, contaminant removal, and subsequent analyses. Taxonomic assignment was performed using the RDP training set version 18 and the “assignTaxonomy” and “addSpecies” functions from the “dada2” package with default parameters. Of the 15,440 amplicon sequence variants (ASVs), 1178 (7.63%) ASVs representing 5.1% of the total read count could be labeled to the species level. Following these data processing steps, the median (first quartile, third quartile) number of reads per sample was 40,585 (24,485–56,360) for PROMISE, 24,433 (14,493–38,585) for ZEBS, and 109,133 (75,901–182,136) for GUMBO. An empirically determined threshold of 1001 reads was used for rarefaction and to remove samples with insufficient reads from further analyses. Rarefied read counts were used for calculation of “Shannon,” “Simpson,” and “Observed” alpha diversity indices using the phyloseq package; relative abundances calculated from the unrarefied data were used for all other analyses. Differences in overall microbiome composition were assessed using permutational multivariate ANOVA (PERMANOVA) with Jenson-Shannon distances. Linear mixed model regression and estimated marginal means were used to identify specific taxa that differed between group variables. A subject-level random effect was included whenever longitudinal samples were used. For genus-level analyses, only taxa present with at least 1% relative abundance in 10% of the samples were tested. For species-level analyses, only taxa present with at least 0.1% relative abundance in 1% of the samples were tested. Beta-binomial regression as implemented in the corncob (v0.4.1) package [27] was also used to identify differentially abundant taxa between group variables. Identical null and non-null overdispersion models were used so as to only identify differentially abundant taxa, and all other parameters were left as default.

All *p* values were corrected for multiple comparisons using the Benjamini–Hochberg FDR method [28], with an adjusted *p* value of 0.05 accepted as statistically significant. All statistical analyses were performed in the R statistical environment (version 4.1.3).

## Results

Following removal of contaminant sequences and samples with low read counts, 687 (641 previously unpublished) of the 756 human milk microbiome samples from 219 of the 224 study participants were evaluable and included in the subsequent analyses as detailed in the study schematic in Fig. 1. Specifically, the numbers of evaluable samples in each cohort were 46 out of 49 in GUMBO, 195 out of 200 in PROMISE maternal ART, 235 out of 264 in ZEBS, and 220 (211 unique) out of 252 in PROMISE transmission.

### GUMBO results: milk microbiome of women with HIV on ART does not differ from women without HIV

**Fig. 2:**
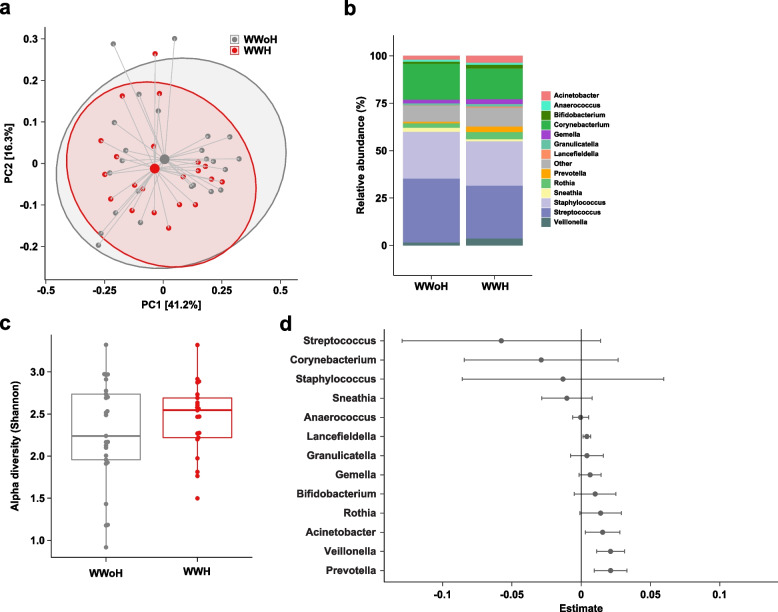
Comparison of human milk microbiome profiles between WWoH and WWH on ART. **a** Principal coordinates analysis of human milk microbiome profiles using Jenson-Shannon divergences. Large points denote centroids and ellipses show 95% confidence areas for the groups as marked. Numbers in brackets denote percent of overall variation explained by each component. **b** Relative abundances at the genus level. **c** Boxplots of alpha diversity using the Shannon metric. **d** Coefficients from linear regression analysis of taxa. Taxa with positive estimates are increased in WWH and taxa with negative estimates are increased in WWoH. Error bars denote 95% confidence intervals

We previously conducted a cross-sectional case–control study of 25 WWH and 25 WWoH in Haiti on the GUMBO study and their infants to examine the effect of maternal HIV infection on the infant microbiome [19]. These women had high CD4 counts, were on ART, and were mostly virologically suppressed. In this prior study, we found no differences in the milk microbiome by maternal HIV status but found differences in the infant gut microbiome. A re-examination of this cohort (see Table 1 for cohort demographics) using newer analytical methods likewise revealed no differences in the milk microbiome by maternal HIV status. As previously observed [19], *Streptococcus* and *Staphylococcus* were the dominant members of the milk community, and maternal HIV infection was not a significant driver of overall microbiome variation (*R*^2^ = 0.003, *p* = 0.896, Table S1 Tab 1). No statistically significant differences in alpha or beta diversity nor in genus- or species-level relative abundances were observed between the WWH and WWoH arms (see Fig. 2 and Tables S1 and S2).

**Table 1:** Baseline characteristics of cohort participants selected for the study


**Variable (mean (SD))**	**WWoH**	**WWH**	***p* Value**
*N*	25	24^a^	
Parity	2.96 (1.90)	3.21 (1.72)	0.634
Days postpartum	71.44 (56.60)	66.88 (45.78)	0.758
Exclusive breastfeeding (%)	19 (76.0)	19 (79.2)	1
CD4 count (cells/mm^3^)	NA	573.79 (275.77)	NA
Plasma HIV-1 RNA (log_10_ copies/mL)	NA	2.31 (1.41)	NA

**Variable (mean (SD))**	**No maternal ART**	**Maternal ART**^b^	***p* Value**
*N*	25	25	
Country (%) ^			
Malawi	16 (64)	16 (64)	
South Africa	7 (28)	7 (28)	
Uganda	1 (4)	1 (4)	
Zimbabwe	1 (4)	1 (4)	
Parity prior to delivery	1.7 (1.3)	1.6 (1.2)	0.91
Gestational age at delivery (weeks)	38.5 (3.3)	38.3 (2.2)	0.81
CD4 count (cells/mm^3^) ^	555 (166)	557 (166)	0.97
Plasma HIV-1 RNA (log_10_ copies/mL) ^	3.85 (0.97)	3.63 (0.86)	0.41

**Variable (mean (SD))**	**CD4** ≤ **200**	**CD4 > 200**	***p* Value**
*N*	20	46	
Site 1 of 2 (%)	13 (65.0)	28 (60.9)	0.967
Parity prior to delivery	2.15 (1.27)	2.24 (1.45)	0.812
Gestational age at delivery (weeks)	38.43 (4.39)	38.57 (3.43)	0.887
Maternal baseline CD4 mean (SD)	151.25 (45.52)	361.93 (115.03)	< 0.001
CD4 < or = 350	20 (100.0)	24 (52.2)	< 0.001
Plasma HIV-1 RNA (log_10_ copies/mL)	5.05 (0.45)	4.76 (0.68)	0.085
Breast milk HIV-1 RNA (1 month)			
Detectable (%)	9/12 (75.0)	14/25 (56.0)	0.451
log_10_ copies/mL (detectable only)	3.27 (1.07)	2.67 (1.00)	0.103

**Variable (mean (SD))**	**Transmitter group**	**Non-transmitter group**	***p* Value**
*N*	22	44	
Parity prior to delivery	2.32 (1.64)	2.16 (1.26)	0.66
Gestational age at delivery (weeks)	38.3 (3.71)	38.7 (3.75)	0.65
CD4 Count (cells/mm^3^) ^	289 (133)	303 (143)	0.71
CD4 < or = 350 (%)	16 (72.7)	28 (63.6)	0.64
CD4 ≤ 200 (%)	7 (31.8)	13 (29.5)	1
Plasma HIV-1 RNA (log_10_ copies/mL) ^	4.83 (0.68)	4.86 (0.61)	0.85
Breast milk HIV-1 RNA (1 week)			
Detectable	5/7 (71.4)	6/11 (54.5)	0.826
log_10_ copies/mL	2.83 (1.13)	2.16 (0.70)	0.132
Breast milk HIV-1 RNA (1 month)			
Detectable	16/22 (72.7)	7/15 (46.7)	0.208
log_10_ copies/mL	3.15 (1.09)	2.43 (0.85)	0.037

**Variable (mean (SD))**	**Transmitter group**	**Non-transmitter group**	***p* Value**
*N*	21^a^	42	
Transmission			
Early mucosal	5		
Breast milk	15		
Unknown	1		
Prophylaxis – maternal ART (%)	7 (33.3)	23 (54.8)	0.181
Maternal ART regimen prior to transmission (%)			0.324
3TC,ZDV,LPV/r	0 (0.0)	3 (7.1)	
FTC,TDF,LPV/r	6 (28.6)	18 (42.9)	
ZDV,sdNVP,FTC,TDF	1 (4.8)	2 (4.8)	
No maternal ARVs	14 (66.7)	19 (45.2)	
Country^ (%)			0.252
India	1 (4.8)	0 (0.0)	
Malawi	9 (47.4)	23 (54.8)	
South Africa	3 (15.8)	9 (21.4)	
Tanzania	1 (4.8)	0 (0.0)	
Uganda	3 (15.8)	7 (16.7)	
Zimbabwe	4 (21.1)	3 (7.1)	
Parity prior to delivery	1.19 (1.12)	2.12 (1.11)	0.003
Gestational age at delivery (wks)	37.99 (2.87)	38.05 (3.10)	0.946
CD4 count (cells/mm^3^) ^	534.38 (188.21)	526.52 (162.35)	0.864
Plasma HIV-1 RNA (log_10_ copies/mL) ^	4.56 (0.74)	4.03 (0.91)	0.026

*WWoH* women without HIV, *WWH* women with HIV, *3tc* lamivudine, *zdv* zidovudine, *lpv/r* ritonavir-boosted lopinavir, *ftc* emtricitabine, *tdf* tenofovir diproxil fumarate, *sd. nvp* single-dose nevirapine

^a^Number of participants selected for the study are shown. Sequences from three participants in the GUMBO WWH and two participants in the PROMISE by Transmission Transmitter Group (one participant each from India and Tanzania) were not evaluable and not included in the final analyses

^b^Maternal antiretroviral therapy (ART) with tenofovir, emtricitabine, and ritonavir-boosted lopinavir

^CD4 count and viral load are from baseline plasma samples

^Study matching criteria as well as date of randomization

Next, we investigated if antiretroviral therapy alters the milk microbiome in WWH.

### PROMISE results: combination ART with TDF/FTC/LPVr is not associated with alterations in the milk microbiome

**Fig. 3:**
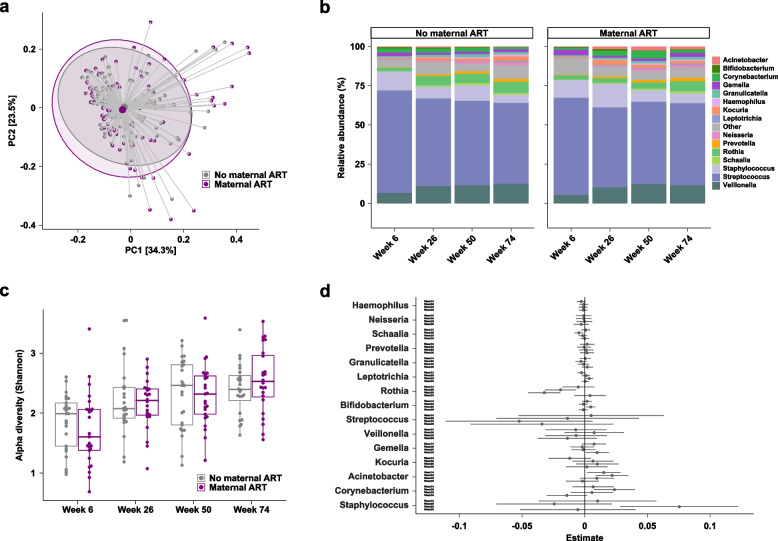
Antiretroviral therapy does not modulate the human milk microbiome. **a** Principal coordinates analysis of human milk microbiome profiles using Jenson-Shannon divergences demonstrates that maternal ART was not a driver of overall microbiome variation (*p* = 0.909). Large points denote centroids, and ellipses show 95% confidence areas for the groups as marked. Numbers in brackets denote percent of overall variation explained by each component. **b** Relative abundances at the genus level stratified by visit. **c** Boxplots of alpha diversity using the Shannon metric. **d** Coefficients from linear regression analysis of taxa stratified by visit. Taxa with positive estimates are increased in mothers on ART, and taxa with negative estimates are decreased in mothers on ART. Error bars denote 95% confidence intervals

The demographics of the 25 women whose infants received nevirapine versus the 25 women who received ART to prevent breastmilk transmission of HIV as part of the PROMISE study are shown in Table 1. Microbiome profiles were obtained for 195/200 samples. Overall, human milk microbiomes were similar between women receiving ART and those not on ART with *Streptococcus* and *Staphylococcus* as the dominant members. PERMANOVA revealed that study visit (6, 26, 50, and 74 weeks), a proxy for duration of lactation, and the microbiome were associated as expected (*R*^2^ = 0.038, *p* < 0.001, Table S1 Table 2), but maternal ART was not a significant driver of overall microbiome variation (*R*^2^ = 0.0009, *p* = 0.909). No statistically significant differences in alpha diversity nor in genus- or species-level relative abundances between the treatment arms were observed at any of the four timepoints (Fig. 3, Table S3).

Since the milk microbiome was not altered in our study of healthy women with HIV nor by maternal antiretroviral therapy, we next investigated whether the milk microbiome differed in women with advanced HIV disease.

### ZEBS results: advanced HIV disease is not associated with alterations in the human milk microbiome

**Fig. 4:**
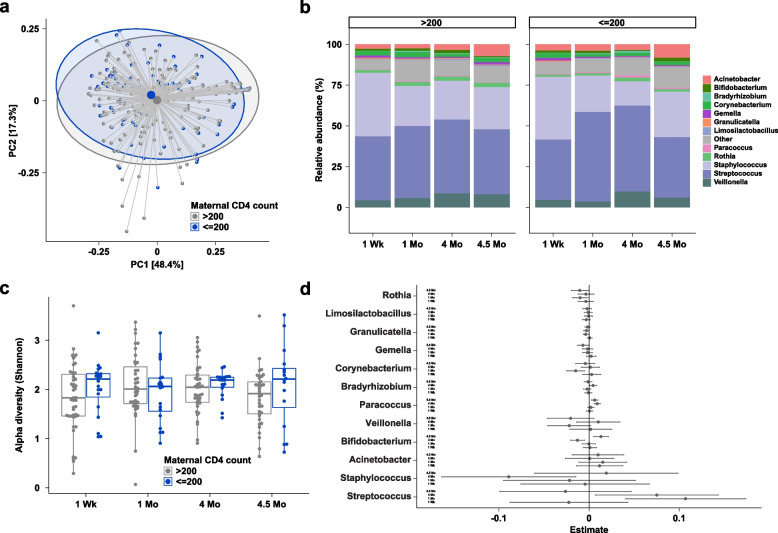
Comparison of human milk microbiome profiles between mothers with high (> 200) and low (≤ 200) CD4 counts. **a** Principal coordinates analysis of human milk microbiome profiles using Jenson-Shannon divergences. Large points denote centroids, and ellipses show 95% confidence areas for the groups as marked. Numbers in brackets denote percent of overall variation explained by each component. **b** Relative abundances at the genus level stratified by visit. **c** Boxplots of alpha diversity using the Shannon metric. **d** Coefficients from linear regression analysis of taxa stratified by visit. Taxa with positive estimates are increased in mothers with low CD4 counts and taxa with negative estimates are decreased in mothers with low CD4 counts. Error bars denote 95% confidence intervals

Using the ZEBS cohort [22], we compared the milk microbiome of mothers with advanced HIV disease (baseline CD4 count < or = 200 cells/mm^3^) versus those with mild disease (baseline CD4 count > 200 cells/mm^3^) who only received a single dose of nevirapine at the time of delivery (see Table 1). PERMANOVA revealed that study visit and microbiota were associated (*R*^2^ = 0.027, *p* < 0.001, Table S1 Tab 3), but maternal CD4 count was not a significant driver of overall microbiome variation (*R*^2^ = 0.003, *p* = 0.263). No statistically significant differences in alpha diversity nor in genus- or species-level relative abundances between women with a baseline CD4 count of < or = 200 versus > 200 cells/mm^3^ were observed at any of the four timepoints (see Fig. 4, Table S4).

Having found no differences in the milk microbiome with HIV infection, antiretroviral therapy, or advanced HIV disease, we hypothesized that perhaps a strongly pro-inflammatory milieu prior to or following transmission of HIV to the infant would be most likely to yield observable effects on the microbiome.

### ZEBS results: early mucosal transmission of HIV is not associated with alterations in the human milk microbiome

We evaluated the milk microbiota of 66 WWH whose infant was either infected with HIV (*N* = 22) or not infected with HIV (*N* = 44) via early mucosal transmission. Although these mothers were matched on baseline maternal CD4 count and *plasma* HIV RNA, the *breast milk* HIV RNA was significantly elevated in cases of transmission at 3.15 versus 2.43 log_10_ copies/mL at 1 month postpartum among 37 evaluable subjects (*p* < 0.05) (see Table 1).

**Fig. 5:**
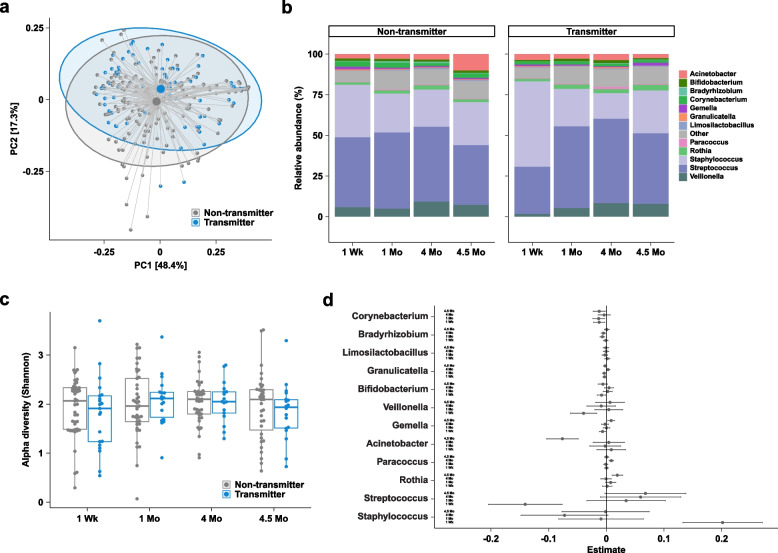
Comparison of human milk microbiome profiles between non-transmitters and early mucosal transmitters. **a** Principal coordinates analysis of human milk microbiome profiles using Jenson-Shannon divergences. Large points denote centroids and ellipses show 95% confidence areas for the groups as marked. Numbers in brackets denote percent of overall variation explained by each component. **b** Relative abundances at the genus level stratified by visit. **c** Boxplots of alpha diversity using the Shannon metric. **d** Coefficients from linear regression analysis of taxa stratified by visit. Taxa with positive estimates are increased in transmitters and taxa with negative estimates are increased in non-transmitters. Error bars denote 95% confidence intervals

The milk microbiome varied with study visit (*R*^2^ = 0.027, *p* < 0.001) and parity (*R*^2^ = 0.006, *p* = 0.041), but not with infant infection (*R*^2^ = 0.003, *p* = 0.197, Table S1 Tab 3). There was no difference in alpha diversity at any timepoint, nor in genus- nor species-level relative abundances in milk samples either before (1 week) or after (1, 4, and 4.5 months) the infant tested positive for HIV (Fig. 5, Table S4). Since there was no observable difference in the milk microbiome in cases of early mucosal transmission, where it is not possible to determine if transmission was intrapartum or through breast milk, we next investigated whether there were differences in the milk microbiome in clear cases of breast milk transmission as well as additional cases of early mucosal transmission in the setting of breast milk prophylaxis from an independent cohort.

### PROMISE results: breast milk transmission and early mucosal transmission of HIV in the setting of antiretroviral prophylaxis is not associated with changes in the milk microbiome

We evaluated the milk microbiota of 61 WWH whose infant was either infected with HIV (*N* = 19) or not infected with HIV (*N* = 42) in the setting of prophylaxis to prevent transmission. Despite matching on CD4 and viral load to the extent possible, plasma viral load was significantly higher in cases where transmission occurred (HIV RNA 4.56 versus 4.03 log_10_ copies/mL, *p* < 0.05). Additionally, parity was lower in women whose infants became infected, 1.26 versus 2.12 prior to delivery (*p* = 0.008). The transmission cases occurred via early mucosal transmission in 5 participants and via breast milk transmission in 13 participants. A negative infant PCR at birth could not be confirmed for a single participant; therefore, *in utero* transmission could not be excluded (see Table 1).

**Fig. 6:**
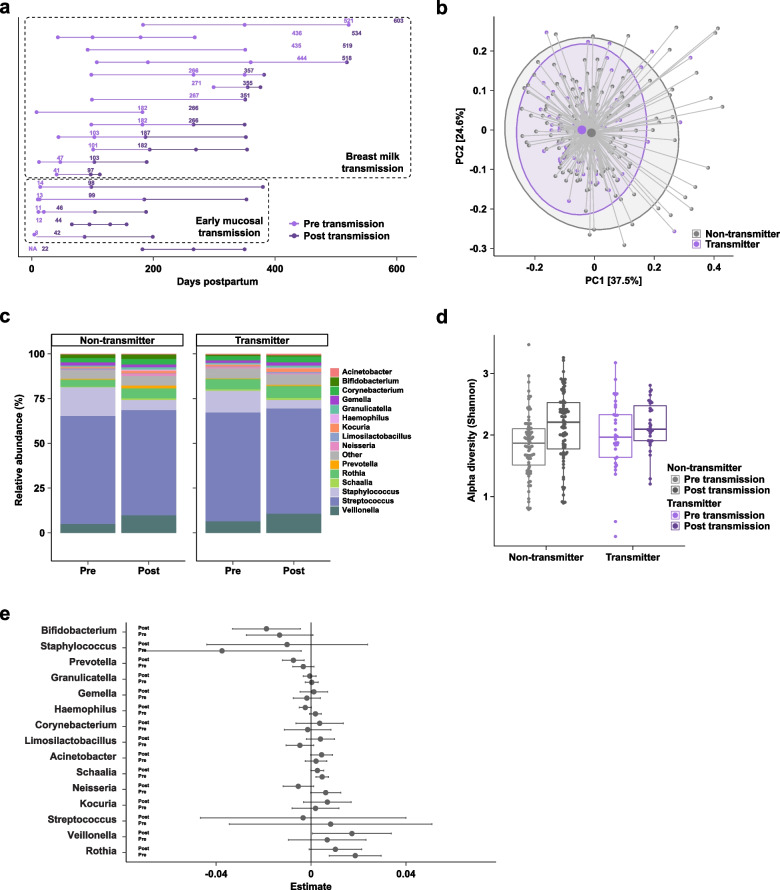
Comparison of human milk microbiome profiles between non-transmitters and transmitters. **a** Schematic of samples collected by postpartum age. Each row represents a single participant. Light and dark purple points denote samples collected prior to (pre) and after (post) transmission, respectively. The two numbers in each row show the age of the infant at the last negative and first positive HIV PCR test (light and dark purple digits, respectively). Dashed boxes mark the early mucosal versus human milk transmitters as indicated. Non-transmitters are not shown in this diagram. **b** Principal coordinates analysis of human milk microbiome profiles using Jenson-Shannon divergences. Large points denote centroids and ellipses show 95% confidence areas for the groups as marked. Numbers in brackets denote percent of overall variation explained by each component. **c** Relative abundances at the genus level stratified by whether the samples were collected pre- or post-transmission. **d** Boxplots of alpha diversity using the Shannon metric. **e** Coefficients from linear regression analysis of taxa stratified by visit. Taxa with positive estimates are increased in transmitters and taxa with negative estimates are increased in non-transmitters. Error bars denote 95% confidence intervals

Days of lactation (age of infant when human milk was sampled) (*R*^2^ = 0.037, *p* < 0.001) and country (*R*^2^ = 0.014, *p* = 0.007) were the main drivers of overall microbiome variation (Table S1 Tab 4). ART regimen also appeared significant on a single PERMANOVA (*R*^2^ = 0.005, *p* = 0.03, Table S1 Tab 4), but this was not observed when the analysis was repeated with non-study regimens removed (*R*^2^ = 0.002, *p* = 0.6, Table S1 Tab 5). Notably, 25 of the women received regimens containing zidovudine (ZDV), all in the first 14 days of lactation. An analysis revealed zidovudine to be the primary driver of this variation (*R*^2^ = 0.012, *p* < 0.001, Table S1 Tab 6). However, this result appears to derive primarily from the timing of zidovudine, as an analysis of regimens restricted to the first 14 days of the study did not find any effect of zidovudine (*R*^2^ = 0.01, *P* = 0.6, Table S1 Tab 7). There was no difference in alpha diversity in samples prior to or following the transmission event, nor in genus- nor species-level relative abundances in milk samples either before or after the transmission event (see Fig. 6, Table S5).

### Combining transmission cohorts and sensitivity analyses

In a combined analysis of both ZEBS and PROMISE transmission cohorts, days of lactation (age of infant when human milk was sampled) (*R*^2^ = 0.016, *p* < 0.001) and parity (*R*^2^ = 0.006, *p* = 0.04) were the main drivers of overall microbiome variation (Table S1 Tab 8). No significant differences in alpha diversity nor in genus- or species-level relative abundances were found in this combined analysis (Figure S1 and Table S6). Sensitivity analyses stratified by mode of transmission, evaluating the PROMISE early mucosal cases alone, the PROMISE breast milk transmission cases alone, and limiting the samples to the time point closest but prior to (immediate pre-sample) and closest but following (immediate post-sample) the transmission event (see Fig. 6, panel a for the schematic of the sample timing) all confirmed these findings, namely, that no robust differences in the milk microbiome were observed either prior to or following the transmission event (see Figures S2–S4, Table S7). Finally, an alternate approach using beta-binomial regression also did not identify any significant differences in genus-level abundances in any of the prior comparisons (Table S8).

## Conclusions

This study evaluates the human milk microbiome of 687 milk samples obtained from 3 distinct cohorts of WWH spanning a 15-year period. We find no difference in the human milk microbiome in WWH on antiretroviral therapy compared to WWoH. Specifically, there was no evidence that the human milk microbiome is altered by HIV infection, by combination ART, by advanced maternal disease, or in cases of infant infection either through early mucosal or breast milk transmission. Our data reveal that the milk microbiome is modulated by parity, geographic region, and duration of lactation with no appreciable signs that HIV infection influences it.

While this case–control study did not find any differences in the milk microbiome in WWH on TDF/FTC/LPVr versus no ART over 74 weeks of lactation, only one ART regimen was specifically tested. We cannot rule out that other regimens may have different consequences. In vitro data suggest that zidovudine and efavirenz may alter the microbiome [14, 16]. Our transmission cohort included 25 samples where the women were on zidovudine, but when compared directly to samples from other WWH in the first 14 days of lactation, no significant differences were seen. Additionally, in a study comparing 24 women randomized to receive no ART versus 25 WWH receiving zidovudine, nevirapine, or lamivudine sampled weekly during the first 4 weeks of lactation, no differences by ART were seen [29]. Taken together, these data are reassuring that no major differences are seen in the milk microbiome with zidovudine. One small study of 17 HEU infants found less *Bifidobacterium* at 6 months postpartum in HEU infant stool versus infants of WWoH and suggested a correlation of lower *Bifidobacterium longum* concentrations with higher breast milk nevirapine concentrations [30]. However, that study compared WWH on ART with WWoH where the WWH breastfed their infants for a shorter duration, and most of the infants were receiving trimethoprim-sulfamethoxazole. Therefore, whether the effects on the infant gut microbiome were attributable to HIV, nevirapine, or a confounder requires further study. In the GUMBO cohort, we found differences in the infant gut microbiome by maternal HIV status in children not receiving trimethoprim-sulfamethoxazole prophylaxis [19]. It is possible that maternal ART transferred to the infant via human milk could alter the infant gut microbiome as some antivirals are secreted in milk at concentrations that may be active [31, 32].

We hypothesized that if HIV infection significantly altered the milk microbiome, and then differences would be seen with advanced maternal disease or potentially in cases of breast milk transmission. However, the milk microbiome appears to remain resilient even in the setting of advanced maternal HIV disease. Additionally, no differences in the milk microbiome were seen in cases of early mucosal transmission or breast milk transmission of HIV. Our study again shows that lower parity and higher viral load are associated with transmission [33, 34].

While our combined data is one of the larger studies of the human milk microbiome with more than 600 samples, particular sub-analyses are limited by sample size. Differences related to breast milk transmission of HIV, either prior to or following the transmission event, may have been masked by the small sample size, the varying length between sample collection and the transmission event, and the highly variable times during lactation when the relatively few transmission events occurred. Additionally, regimen-specific alterations of the human milk microbiome may have been missed as this study only had sufficient samples numbers to assess TDF/FTC/LPVr, and none of the antiretrovirals in this regimen are known to have in vitro antibacterial activity. Moreover, some of the milk samples in the PROMISE cohort were kept cold for up to 6 h prior to being stored at − 70 °C. If some bacterial overgrowth occurred during this transition, it may have altered the taxonomic profile of the samples. A final caveat of our study is the limited taxonomic resolution of the amplicon-based approach using the 16S V4 region. It is possible that HIV- and/or ART-related perturbations occur at the species or even strain level; further studies utilizing other methods with robust species-level resolution would be needed to detect these differences in the milk microbiome.

The milk microbiome changes over the course of lactation, by parity and by region; however, we find no evidence that the human milk microbiome is altered by maternal HIV infection, disease severity, or HIV treatment. Our investigations of the milk microbiome do not explain the immunologic abnormalities, stunting, and possible cognitive differences experienced by children born to WWH. Further study is warranted to examine regimen-specific effects or potential effects of maternal ART on the infant gut microbiome. Additionally, investigations into other potential mechanisms to understand why the approximately one million infants born to women with HIV that escape infection, but do not escape harm, are urgently needed [35].

## Supplementary Information

Supplementary Material 1: Figure S1. Comparison of human milk microbiome profiles between non-transmitters and transmitters combining both ZEBS and PROMISE cohorts. (a) Principal coordinates analysis of human milk microbiome profiles using Jenson-Shannon divergences. Large points denote centroids and ellipses show 95% confidence areas for the groups as marked. Numbers in brackets denote percent of overall variation explained by each component. (b) Relative abundances at the genus level stratified by whether the samples were collected pre- or post-transmission. (c) Boxplots of alpha diversity using the Shannon metric. (d) Coefficients from linear regression analysis of taxa stratified by visit. Taxa with positive estimates are increased in transmitters and taxa with negative estimates are increased in non-transmitters. Error bars denote 95% confidence intervals.

Supplementary Material 2: Figure S2. Comparison of human milk microbiome profiles stratified by mode of transmission. (a) Principal coordinates analysis of human milk microbiome profiles using Jenson-Shannon divergences. Large points denote centroids and ellipses show 95% confidence areas for the groups as marked. Numbers in brackets denote percent of overall variation explained by each component. (b) Relative abundances at the genus level stratified by pre- versus post-transmission timepoints. (c) Boxplots of alpha diversity using the Shannon metric. (d) Coefficients from linear regression analysis of taxa stratified by timing of sample. The specific comparisons are indicated in text along the sides. Error bars denote 95% confidence intervals.

Supplementary Material 3: Figure S3. Comparison of human milk microbiome profiles between non-transmitters and transmitters at visits immediately prior to and following transmission. (a) Principal coordinates analysis of human milk microbiome profiles using Jenson-Shannon divergences. Large points denote centroids and ellipses show 95% confidence areas for the groups as marked. Numbers in brackets denote percent of overall variation explained by each component. (b) Relative abundances at the genus level stratified by pre- versus post-transmission timepoints. (c) Boxplots of alpha diversity using the Shannon metric. (d) Coefficients from linear regression analysis of taxa stratified by timing of sample. Error bars denote 95% confidence intervals.

Supplementary Material 4: Figure S4. Comparison of human milk microbiome profiles between non-transmitters and transmitters at visits immediately prior to and following transmission. (a) Relative abundances at the genus level for each sample in the groups as indicated. (b) Principal coordinates analysis of human milk microbiome profiles using Jenson-Shannon divergences. Large points denote centroids and ellipses show 95% confidence areas for the groups as marked. Numbers in brackets denote percent of overall variation explained by each component. (c) Boxplots of alpha diversity using the Shannon metric. (d) Coefficients from linear regression analysis of taxa stratified by timing of sample. Error bars denote 95% confidence intervals.

Supplementary Material 5: Table S1. PERMANOVA results for each comparison as indicated. 

Supplementary Material 6: Table S2. Statistical comparisons of alpha diversity and genus- and species-level relative abundances for the GUMBO cohort, comparing WWH to WWoH.

Supplementary Material 7: Table S3. Statistical comparisons of alpha diversity and genus- and species-level relative abundances for the PROMISE cohort, comparing the maternal ART arm to the no ART arm. 

Supplementary Material 8: Table S4. Statistical comparisons of alpha diversity and genus- and species-level relative abundances for the ZEBS cohort. 

Supplementary Material 9: Table S5. Statistical comparisons of alpha diversity and genus- and species-level relative abundances for the PROMISE cohort, comparing cases of early mucosal transmission and breast milk transmission to non-transmissions.

Supplementary Material 10: Table S6. Statistical comparisons of alpha diversity and genus- and species-level relative abundances for the combined ZEBS and PROMISE cohorts. 

Supplementary Material 11: Table S7. Sensitivity analyses stratifying by mode of transmission (stratified-), limiting the samples to the time point closest but prior to (immediate pre-sample) and closest but following (immediate post-sample) the transmission event (at transmission-), evaluating the PROMISE early mucosal transmission cases alone (EMT-), and the PROMISE breast milk transmission cases alone (BMT-). ‘adiv’ denotes that alpha diversity metrics are shown on that tab for the noted comparison. 

Supplementary Material 12: Table S8. Beta-binomial (‘corncob’) regression demonstrating no significant differences in genus-level abundances in any of the prior comparisons.

## Data Availability

Sequence data have been deposited in the NCBI Sequence Read Archive under BioProject accessions PRJNA1073967 (for ZEBS and PROMISE) and PRJNA305167 (for GUMBO). All analysis code is available at https://github.com/AldrovandiLab/Milk_Microbiome/.

## Abbreviations

3TC: Lamivudine
ART: Antiretroviral therapy
ARV: Antiretrovirals
CHEU: Children whose mothers had HIV infection (“exposed”) but not infected with HIV
CHUU: Children whose mothers did not have HIV infection (“not exposed”) and not infected with HIV
FTC: Emtricitabine
GUMBO: GUt Microbiome, Breastmilk and Oligosaccharides Study
HEU: HIV-exposed uninfected
HIV: Human immunodeficiency virus
HUU: HIV-unexposed uninfected
LPV/r: Ritonavir-boosted lopinavir
PROMISE: Promoting Maternal Infant Survival Everywhere Study
SD: Standard deviation
SD NVP: Single-dose nevirapine
TDF: Tenofovir diproxil fumarate
WWH: Women with HIV
WWoH: Women without HIV
ZDV: Zidovudine
ZEBS: Zambia Exclusive Breastfeeding Study
